# Inequity in Timeliness of MMR Vaccination in Children Living in the Suburbs of Iranian Cities

**Published:** 2015-06

**Authors:** Rahmatollah Jadidi, Abolfazl Mohammadbeigi, Narges Mohammadsalehi, Hossein Ansari, Ebrahim Ghaderi

**Affiliations:** 1 Department of Education Development Center (EDC), Arak University of Medical Sciences, Arak, Iran;; 2 Assistant professor, PhD in Epidemiology;; 3 Health policy and promotion Research Center, Department of epidemiology and biostatistics, Qom University of Medical Sciences, Qom, Iran;; 4 Health Promotion research Center, Department of Epidemiology and biostatistics, Zahedan University of Medical Sciences, Zahedan, Iran;; 5 Assistant professor, PhD in Epidemiology, Social Determinants of Health Research Center, Kurdistan University of Medical Sciences, Sanandaj, Iran

**Keywords:** Inequity, Disparity, Timeliness, Immunization, Delay Vaccination, MMR

## Abstract

**Introduction:**

High coverage of immunization is one of the indicators of good performance of health system but timely vaccination is another indicator which is associated with protective effect of vaccines. The present study aimed at evaluating the inequity in timely vaccination with a focus on inequities in timeliness by gender, birth order, parents’ education and place of residence (rural or urban).

**Methods:**

A historical cohort study was conducted on children of 24-47 months of age who were living in the suburbs of big cities in Iran and were selected through stratified proportional sampling method. Only children who had vaccine cards -i.e. 3610 children -were included in data analysis. The primary outcome was age-appropriate vaccination of MMR1. Inequity was measured by Concentration Index (C) and Relative Index of Inequity (RII). Inequity indexes were calculated according to the mother and father’s education, child birth order, child’s sex and the family’s place of residence at the time of vaccination.

**Results:**

The overall on-time MMR1 vaccination was 70% and 54.4% for Iranians and Non-Iranians, respectively. The C index of mother and father’s education for timely MMR vaccination was 0.023 and was 0.029 in Iranian children as well as 0.044 and 0.019 for non-Iranians, respectively. The C index according to child order in Iranians and Non-Iranians was 0.025 and C=0.078. With regard to children who lived in cities, the on-time vaccination was 0.36% and 0.29% higher than that in rural areas . In male children it was 0.12% and 0.14% higher than that in female children for Iranians and Non-Iranians, respectively.

**Conclusion:**

Timeliness MMR vaccination in Iranian children is higher than that in non-Iranian children. Regarding the existence of differences in timely vaccination rate in all Iranian and Non-Iranian children, no evidence was observed for inequity by focusing on parents’ education, birth order, gender or place of residence. So, increasing timeliness of vaccination for enhancing the protective effect of vaccines can be considered a health-related goal in Iran after receiving high immunization coverage.

## INTRODUCTION

Childhood immunization is an investment for protection against some non-communicable (1, 2) as well as infectious diseases. It is reported that timely vaccinations in Measles, Mumps and Rubella (MMR) vaccine is related to the decreased risk of childhood asthma in 7-year-old children (1, 3). Performance of immunization programs is measured by immunization coverage in children at certain predefined ages, typically reported at 12 months or 24 months of age, as the most widely used vaccine indicator (4, 5). High coverage of immunization is one of indicators of good performance of health system but the timely vaccination is another indicator that is associated with the protective effect of vaccines (5, 6).

Based on the recent World Health Organization (WHO) assessment as well as the estimates of ministry of health and medical education of Iran, 95 to 98% of the children younger than six years of age were immunized in 2004 (7). In addition, recently our work showed that the immunization coverage in suburbs of Iranian big cities is higher than 97% (8). The vaccination coverage in infants is 90% or higher, which is a prerequisite for control of vaccine-preventable infectious diseases but for the elimination of measles and rubella an even higher coverage is required (5). Although vaccination in Iran is mandatory for school registration and can be received in all rural and urban areas, a proportion of children receive the vaccines with delay (7). Timely vaccination has critical importance for reducing disease risk due to enlargement of gap between losing the protection of maternal antibodies and vaccine-induced protection (2, 9). So, vaccination delay can potentially have severe consequences (2, 3). The recommended age for the first dose of Measles, Rubella and Mump (MMR1) in Iran is at 12 month of age (10). Since maternal immunity remains nine months to one year after birth in the child, the delay in MMR vaccination can expose the child to measles agent (2, 9).

Previous studies have shown that vaccination status is associated with some family characteristics such as race, ethnicity, economic status and the education level of parents as well as some factors such as child rank or gender (1, 3, 5, 11-13). However, the generalizability of these factors for all people can create biased decisions. In addition, other studies showed inequity was related to gender or place of residence and other socioeconomic variables (14, 15). Also, it was not unclear whether the difference level of these variables means there are evidences of inequity regarding these socio-demographic variables. As such, the current study aimed at determining the risk factors of MMR vaccination delay and estimating the inequity related to parents’ education, child gender and order as well as place of residence at vaccination time.

## MATERIALS AND METHODS

In a historical cohort study the children aged 24-47 months and living in the suburbs of five big cities of Iran (Tehran, Esfahan, Arak, Mashhad and Zahedan) were surveyed in June 2013. In each city, the areas with the most concentration of migrants (Iranian and non-Iranian) and with low socio-economic criteria were listed based on the information provided by local health authorities. The survey included the immunization cases based on immunization card plus history of vaccination according to the mother’s recall memory. However, only children with vaccination cards were included in the inequity analysis.

The stratified proportional sampling method was used for each city based on latest census for each defined area that had been described in the previously mentioned phase. Clusters were defined as any area highlighted by local health officers based on lower social class, population density of low health indicators, and high density of migrants. All suburban areas for each city were selected to minimize the sampling errors. The average size of clusters was estimated according to local authorities’ information and existing documents for all the cities under the study. Cities which had higher population included higher sample in the survey. Only children with valid date of vaccination (with written documentations such as vaccination card) were included in data analysis. So, out of 4502 children interviewed only 3610 children had vaccination cards and consequently were included in data analysis. The percentages of subjects from Tehran, Isfahan, Mashhad, Arak and Zahedan was 44.9, 27.7, 19.9, 4.1 and 3.7, respectively. More details is presented in our recent work.[16]

### Data gathering and data analysis

The required data were collected using a prepared questionnaire by house-to-house interview method. The questionnaire was a standard instrument designed according to the questionnaire offered by WHO for the estimation of vaccination coverage (9). Interviewers in this study were trained university students. The questionnaires were completed based on the vaccination card information. The study protocol was approved by the consent of the ethical committee of Arak University of Medical Sciences. Moreover, informed consent was taken from mothers of each study cases before administration of the questionnaire.

Our outcome in current study was age appropriate vaccination of MMR1. Because MMR is a long established vaccine with one dose in the first 2 years of life and injected at 12 month years of age, delay in this dose was evaluated. Delay in age appropriate vaccination is defined if the MMR vaccination is conducted one week after the recommended age for MMR. Different studies have applied the first 7 days after the recommended age as delay (5, 13), but delay in vaccination can be defined by sensitivity of surveillance system. Due to the high immunization coverage in Iran (4, 17), the 7 days after recommended age is regarded as delay.

### Statistical analysis

Data collection was conducted in one month and data were analyzed by stata and Excel softwares. The primary outcome of study was the first age-appropriate dose of MMR immunization defined as the reception of that vaccine until one week after the first birthday. Therefore, on-time vaccination was defined as If a child had received the first dose of MMR vaccine with more than one week delay after the exact date. Hence that child was classified as immunized with delay.

Descriptive statistical method was used to estimate the prevalence of on-time MMR vaccination in the studied areas. Chi square test was used for comparing on-time MMR vaccination prevalence between the two sexes and among people with different places of residence at the time of child vaccination. Inequity was measured by Concentration Index (C) and Relative Index of Inequity (RII). Inequity indexes were calculated according to the mother and father’s education, child birth order, child’ gender and family’s place of residence at the time of vaccination. For ranking variables as parents’ education (Illiterate, Elementary school, Guidance school, High school and college), child birth order (1st child, 2nd child, 3rd child and 4th child or higher) C index was calculated and for child’s sex (male or female) and family’s place of residence at the time of vaccination (rural or urban) the RII was estimated (13). The C index is common inequity measure for health outcomes which has been used continually in recent studies (18-20). The C and it’s confidence interval were calculated by the Kakwani *et al*. formula and the value of C varied between -1 to +1 (20, 21). The RII was calculated as the proportion of Slope Index of Inequity (SII) by the of on-time vaccination percent in the studied people. It should be mentioned that the SII in RII formula is the regression coefficient (β). Binary logistic regression was used to calculate the β for sex and place of residence.

## RESULTS

The overall on-time MMR1 vaccination was 68.2% (2430/3564). The on-time MMR1 vaccination for Iranian and non-Iranian children was 70% and 54.4%, respectively. According to Table 1, there was a significant difference on prevalence of on -time MMR vaccination among different levels of education regarding Iranian parents (*p*<0.001), but this difference was not observed among non-Iranian parents (*p*>0.05). Based on our results, the C index for mothers’ education as an inequity index of on-time MMR vaccination among Iranian and non-Iranian people was C=0.023, CI95%: -0.034 to 0.029 and C=0.044, CI95%: -0.16 to 0.104. This index for fathers’ education among Iranian and non-Iranian people was 0.029 (-0.028, 0.086) and 0.019(-0.143, 0.181), respectively. Also, as indicated in Table 2, there was no significant inequity in C index according to child order in Iranian (C=0.025, CI95%: -0.067 to 0.017) and non-Iranian people (C=0.078, CI95%: -0.175 to 0.019). Furthermore, the prevalence of on-time MMR vaccination in the first child of Iranian and Non-Iranian people was 0.29% and 0.53% higher than those in the fourth child and children born after (RII=0.029 and 0.053),

**Table 1:** The prevalence, Concentration Index (C) with 95% Confidence interval for C and relative index of inequity for on-time MMR vaccination by different level of mothers and fathers’ education

Educational level	Mothers		Fathers	
	Iranian	Non-Iranian	Iranian	Non-Iranian

IL literature	55.9 (71/127)	50.2 (116/231)	47.6 (40/84)	52.7 (96/182)
Elementary school	65.1 (363/558)	59.8 (61/102)	65.6 (389/593)	55.4 (72/130)
Guidance school	70.2 (424/604)	55.6 (30/54)	69.5 (598/860)	50.7 (34/67)
High school	73.1 (1017/1391)	63.3 (19/30)	73.2 (806/1101)	62.1 (18.29)
College	70.6 (324/459)	100 (2/2)	73 (362/496)	77.8 (7/9)
Total	70.1 (2199/3139)	54.4 (228/419)	70 (2195/3134)	54.4 (227/417)
Chi-square *P* Value	<0.001	0.240	<0.001	0.415
C (Confidence Interval 95% C)	0.023 (-0.034 , 0.080 )	0.044 (-0.160 , 0.248 )	0.029 (-0.028 , 0.086 )	0.019 (-0.143 , 0.181)
Relative index of Inequity	-0.21	-0.35	-0.25	-0.191

**Table 2:** The prevalence, Concentration Index (C) with 95% Confidence interval for C and relative index of inequity for on-time MMR vaccination by birth order of children

Birth order	On-time vaccination -Iranian	On-time vaccination in Non-Iranian children

1^st^ child	72.9 (922/1264)	64.6 (42/65)
2^nd^ child	70(916/1308)	62.2 (79/127)
3^rd^ child	66.2 (272/411)	48.8(42/86)
4^th^ child or above	56.1 (87/155)	46.1(65/141)
Total	70(2197/3138)	54.4(228/419)
Chi-square *P* value	<0.001	0.013
C (Confidence Interval 95% C)	-0.025 (-0.067, 0.017)	-0.078 (-0.175,0.019)
Relative index of Inequity	0.29	0.53

According to Table 3, Chi square test did not show any significant difference regarding on-time vaccination prevalence between child’s sex in Iranian and Non-Iranian people (*p*>0.05). The place of residence had more effect on dispartity regarding on-time vaccination than that of child’s sex among Iranian and non-Iranian people. Our results indicated that in the Iranian and non-Iranian children who lived in cities, the on-time vaccination is 0.36% and 0.29% higher than that in rural areas (RII=0.36 and 0.29), respectively. In addition, the results showed that there was a little disparity in child’s sex among Iranians and Non-Iranians. The prevalence of on-time vaccination for male children in Iranian and Non-Iranian were 0.12% and 0.14% higher than those for female children (RII=0.12 and 0.14), respectively.

**Table 3:** The prevalence and relative index of inequity of on-time MMR vaccination by prior place of residence and child’s sex

On-time vaccination	Variables		Prevalence of on-time vaccination	*P* value	Relative index of Relative Index of Inequity

Iranian children	Prior place of residence	City	70.2 (2116/3016)	0.283	0.36
		Rural	64.6 (51/79)		
		Total	70 (2167/3095)		
	Child’s Sex	Female	69.1 (1059/1532)	0.296	0.116
		Male	70.8 (1139/1608)		
		Total	70 (2198/3140)		
Non-Iranian children	Prior place of residence	City	55.1 (199/361)	0.634	0.29
		Rural	51.2 (21/41)		
		Total	54.7 (220/402)		
	Sex	Female	53.4 (102/191)	0.703	0.138
		Male	55.3 (126/228)		
		Total	54.4 (228/419)		

## DISCUSSION

The results showed that there was no evidence of inequity in timely vaccination on the basis of education of parents, birth order and gender of child as well as place of residence at the time of vaccination. In addition, the overall timely MMR1 vaccination was 70% for Iranian whereas this prevalence was 54.4% for Non-Iranian children. In the study by Senessie *et al* in Sierra Leonean (22), the age-inappropriate immunization was 29%, which was similar to our results. According to results, there was a statistical significance in rate of timely vaccinations based on parents’ education in Iranian children. Moreover, there was a significant difference on timely vaccination in children with different birth orders in Iranian and Non-Iranian people. Other studies have also shown that socio-demographic factors such as mother’s education are related to timeliness or delay in vaccination (3, 15, 23, 24).

From the viewpoint of health policy and inequity issues, no evidence of inequity was found in the timely vaccination in studied children based on nationality. Maternal illiteracy is one of the most significant factors that revealed a most important contribution in delay of vaccination or immunization coverage in studies after decomposition inequity analysis (15, 23, 25). In addition, socioeconomic status or wealth index is one of the most important factors related to immunization coverage inequity. However, the parents’ education can be a surrogate variable in Iranian society describing a high percentage of variance of wealth index after factor analysis (26). Needless to say that in our recent study, parents’ education did not show evidences of inequity in the access and utilization of oral health care (21).

Although a significant difference in timeliness of MMR vaccination observed between male and female children but no sex inequity is found. The same results have been found in other study (21). Corsi *et al* showed that birth order and sex of children are related factors of inequity in immunization coverage, but the concentration index for birth order and child gender in our study was very low and insignificant (14). Also, the education -related inequity based on C index for mothers and fathers of Iranian children was 0.023 and 0.029, respectively while this index for oral health care utilization (OHCU) was calculated to be 0.097 and 0.091 (21). Moreover, this index showed that the parents’ education related to immunization inequity is very lower than that in OHCU. However, vaccination is a free health care in contrast with oral care which is expensive. In addition to educational level of parents, other studies have also shown that people with higher wealth index were significantly more likely to fully immunize their children (23). The highest C index was calculated for child order in non-Iranian people. However, since the inequity measure in our study was not statistically significant based on CI of C, decomposition of C was meaningless.

However, according to literature, the immunization coverage in Iran is high in all different levels of people (16, 27, 28). In addition, it is shown that timely vaccination is an important component of infection control by reducing transmission among susceptible populations (14). Therefore, countries with high levels of vaccination coverage such as Iran should encourage people for immunization at the earliest appropriate age as an important public health goal (8). Also, it is observed that public health programs can be helpful in reducing the gender and socio-demographic inequities in the whole of people (29) as the EPI caused removing disparities In Iran and other places (30, 31). Nevertheless, this study was the first national study in outskirt of Iranian cities regarding to inequity in vaccination and other studies suggested finding the coverage and on-time vaccination in immigrants to Iran. In addition, the analysis only included the children who had vaccine cards due to the delay time in children without card was unknown. Moreover, National surveillance of age-appropriate vaccination is suggested to identify sub-groups of populations with the high prevalence of vaccination delay and related factor assessments of timely vaccination (3).

## CONCLUSION

Regarding to the existence of differences in timely vaccination rate according to sex and nationality but no evidence was observed for inequity by focusing on parents’ education, birth order, gender, or place of residence. All children with different levels of parents’ education and birth order, the two genders, rural and urban places of residence have an equal age- appropriate immunization in MMR. Therefore, it seems that increasing timeliness of vaccination for improving the protective effect of vaccines can be considered a health-related goal in Iran after receiving high immunization coverage.
